# Classifying retinopathy of prematurity

**Published:** 2017

**Authors:** Andrea Molinari, Dan Weaver, Subhadra Jalali

**Affiliations:** Pediatric Ophthalmologist: Hospital Metropolitano, Av. Mariana de Jesus Oe-8, Quito, Ecuador.; Pediatric Ophthalmologist: Billings Clinic, Billings, Montana, USA.; Director: Newborn Eye Health Alliance, (NEHA) and Director, Quality: LV Prasad Eye Institute, Hyderabad, India.

**Figure:**
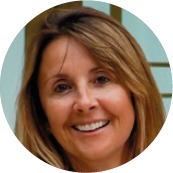
Andrea Molinari

**Figure:**
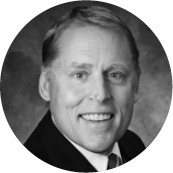
Dan Weaver

**Figure:**
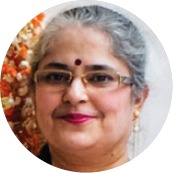
Subhadra Jalali

**Knowing how to classify retinopathy of prematurity is essential as it provides information on the prognosis and guides decision making about screening and treatment.**

It is important to classify retinopathy of prematurity (ROP) in each eye, at each screening session. Doing so makes it possible to screen babies consistently and to make decisions about whether further screening is required and when, or whether laser treatment or surgical management is needed. The International Committee for the Classification of ROP^1^ has classified it using the following criteria:

1. The severity of the ROP
2. The zone in the retina where ROP is found
3. The extent of the ROP
4. Whether the retinal blood vessels are dilated and/or tortuous (pre-plus or plus disease)
5. Whether aggressive posterior ROP is present

## The severity of the ROP

ROP can develop when the immature retinal blood vessels have not reached the edge of the retina, known as the ora serrata.

**Figure 1:**
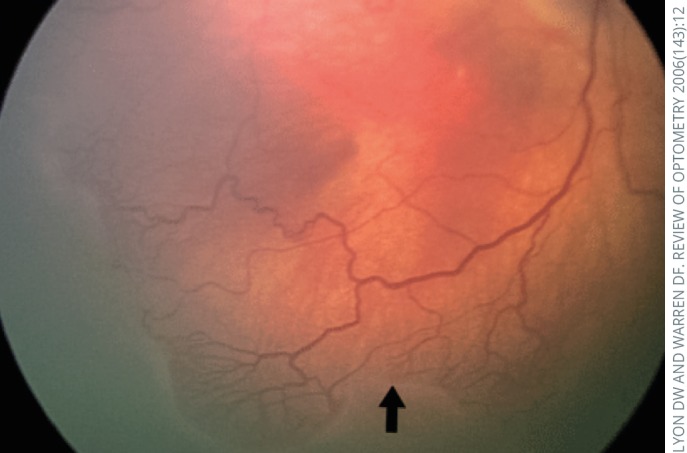
Stage 1 ROP: Demarcation line (arrow)

**Figure 2:**
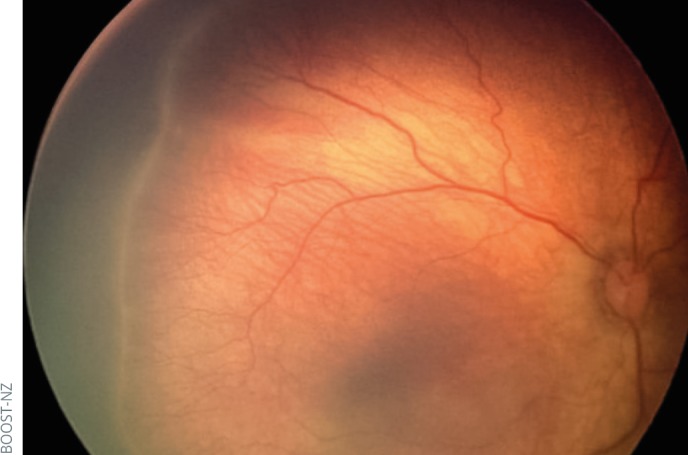
Stage 2 ROP: The demarcation line becomes a ridge with both height and width

**Figure 3:**
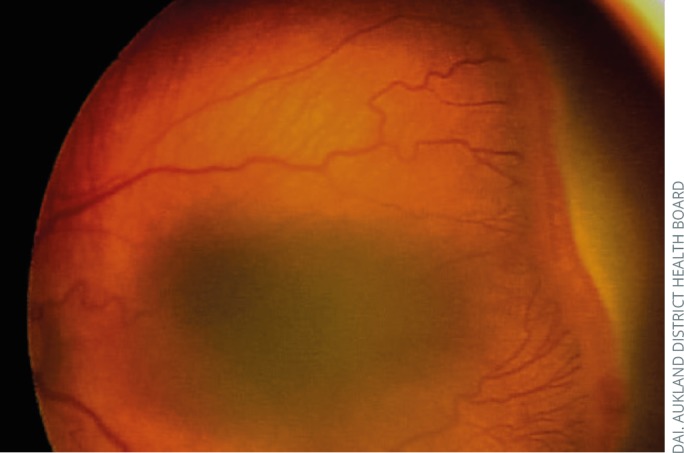
Stage 3 ROP: Abnormal blood vessels grow and multiply within the ridge

**Figure 4:**
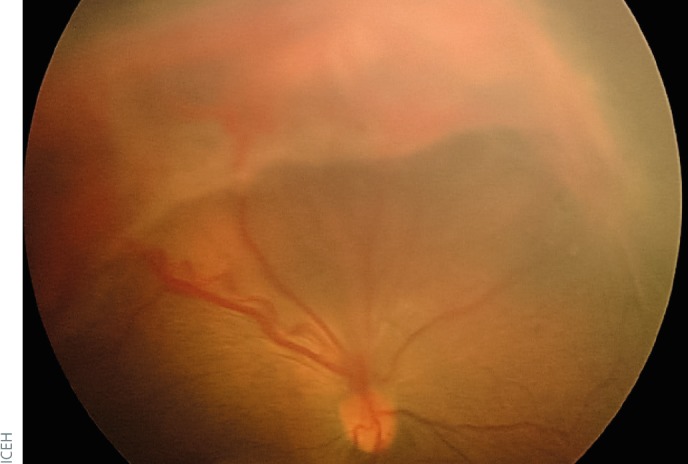
Stage 4 ROP: Sub-total retinal detachment

**Figure 5:**
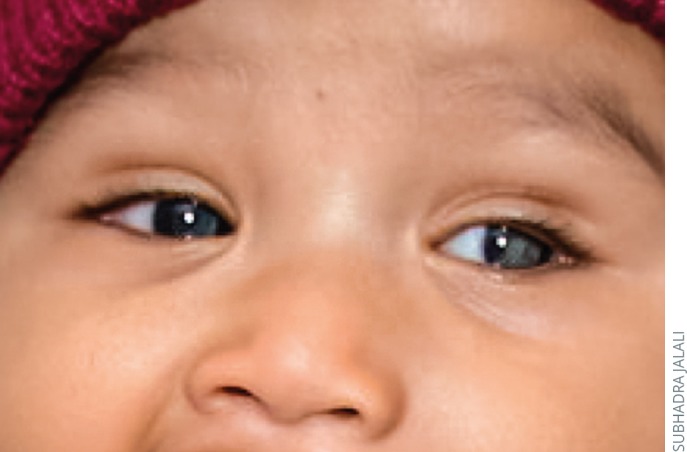
Stage 5 ROP: Total retinal detachment. Parents may notice this as something white in the eyes

- **Stage 1 ROP: Demarcation line.** A whitish line is visible between the normally vascularised retina and the peripheral retina in which there are no blood vessels (Figure 1)
- **Stage 2 ROP: Visible ridge.** The demarcation line develops into a ridge, with height and width, between the vascular retina and peripheral retina (Figure 2).
- **Stage 3 ROP: Blood vessels in the ridge.** Blood vessels grow and multiply (proliferate) and are visible in the ridge (Figure 3).
- **Stage 4 ROP; Sub-total retinal detachment.** Vitreoretinal surgery may be indicated (Figure 4).
- **Stage 5 ROP: Total retinal detachment.** No treatment is usually possible (Figure 5).

**Figure 6:**
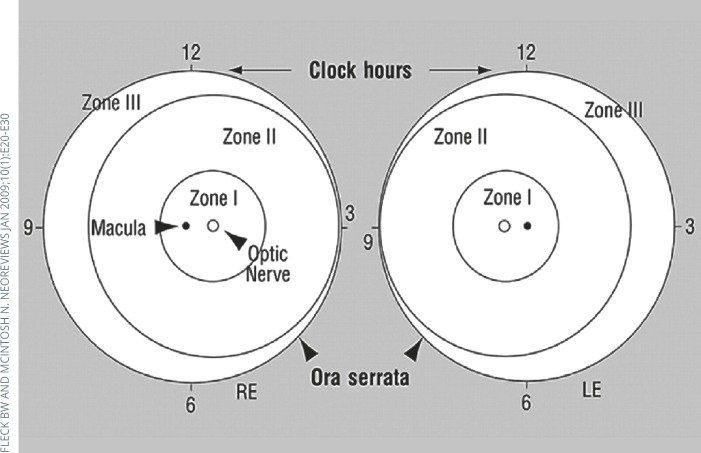
The three zones of ROP

## The zones in the retina where ROP is found

The three zones of ROP are centred on the optic disc (Figure 6).

- Zone I is the small circle of retina around the optic disc. The radius of the circle is twice the distance from the macula to the centre of the optic disc
- Zone II is the ring-shaped section of the retina surrounding zone I, which extends to the ora serrata on the nasal side
- Zone III is a crescent-shaped area of temporal retina.

ROP in zone I is more likely to progress and become severe than ROP in zones II or III.

## The extent of the ROP

The extent of disease is recorded as clock hours, in twelve 30° or 1-hour sections (Figure 6). The clock hours recorded are the total clock hours involved, not just the contiguous sectors.

## The presence of plus disease

**Figure 7:**
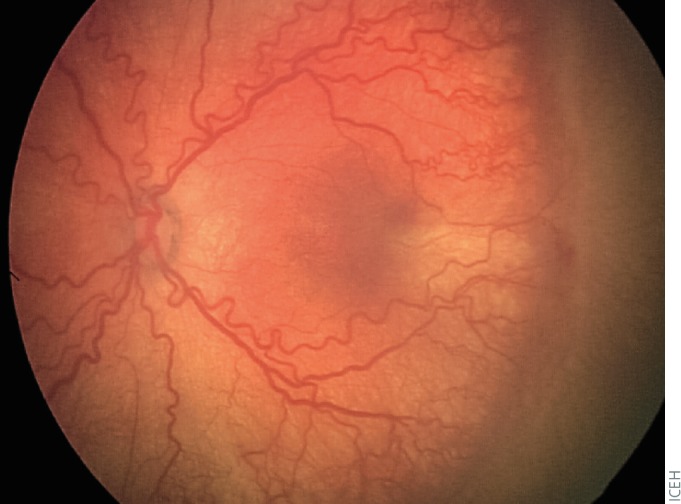
Plus disease: dilated and torturous veins

In plus disease, retinal arterioles and venules near the optic disc are dilated and tortuous. In pre-plus disease the changes are less pronounced, or may not affect all the blood vessels (Figure 7).

## The presence of aggressive posterior ROP (AP-ROP)

**Figure 8:**
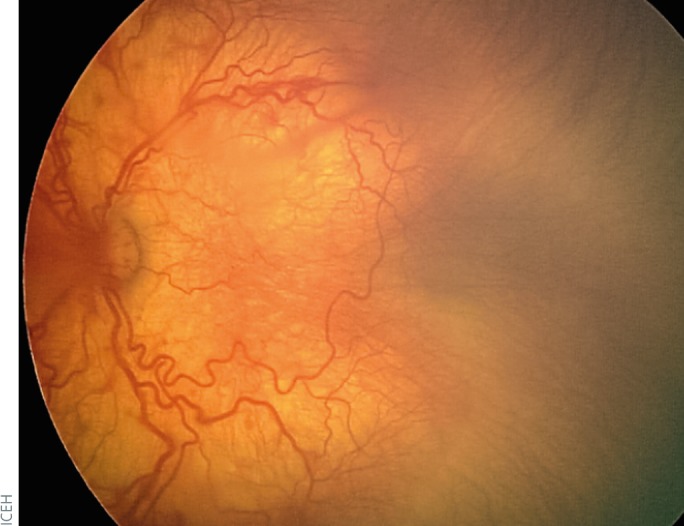
Aggressive posterior ROP (in zone I)

Aggressive posterior ROP (AP-ROP) is nearly always in zone I. The proliferating blood vessels are flat and difficult to see, and plus disease is always present (Figure 8).

**NOTE:** It is very important to recognise AP-ROP as it can progress extremely quickly to retinal detachment. Treatment should be given within 48 hours.

## How the classification can be used

Classification of ROP guides decision making about screening and treatment. For example:

- If immature retinal vessels are present, screening should be repeated
- If ROP is in zones II or III (further away from the optic disc) and is at stage 1 or 2, without any plus disease, the prognosis is good and the ROP is likely to resolve without treatment. Repeat screening is required in 1–2 weeks.
- If ROP is in zone I, or if it is Stage 3 with plus disease, or aggressive posterior ROP is present, urgent treatment is needed as the disease is very likely to progress to retinal detachment.

## Scarring after ROP

**Figure 9:**
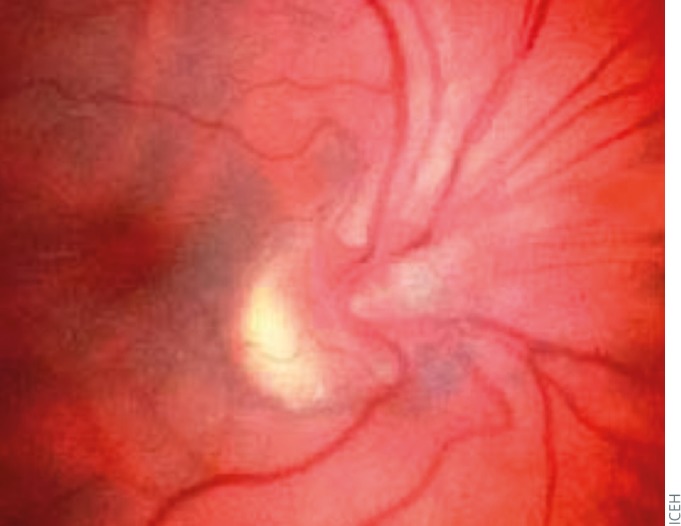
Scarring after ROP

Untreated ROP can sometimes heal with scarring in the peripheral retina and vitreous. This distorts the retina, leading to macular dragging or retinal folds. These signs are not included in the International Classification of ROP, but can be associated with loss of vision (Figure 9).
